# Food preparation skills and obesity risk in European children aged 6–9 years: a cross-sectional study using WHO COSI 2022–2024’

**DOI:** 10.1007/s00394-026-03928-6

**Published:** 2026-02-28

**Authors:** Karen L. Vaughan, Marta Buoncristiano, Julienne Williams, Vessel Duleva, Tatjana Hejgaard, Kitti Susovits, Shynar Abdrakhmanova, Ausra Petrauskiene, Maria-Victoria Racu, Igor Spiroski, Marion M. Hetherington, Janet E. Cade

**Affiliations:** 1 University of Leeds, Leeds, UK; 2 World Health Organization, Geneva, Switzerland; 3 National Center of Public Health and Analyses, Sofia, Bulgaria; 4 Danish Health Authority, Copenhagen, Denmark; 5 National Centre for Public Health and Pharmacy, Budapest, Hungary; 6 National Scientific Center for Healthcare Development (named after S. Kairbekova), Astana, Kazakhstan; 7 Lithuanian University of Health Sciences, Kaunas, Lithuania; 8 National Agency for Public Health, Chisinau, Republic of Moldova; 9 Institute of Public Health/Faculty of Medicine, Ss. Cyril and Methodius University, Skopje, North Macedonia

**Keywords:** Food preparation skills, Childhood obesity, Nutrition education, Fruit and vegetable intake, Public health nutrition

## Abstract

**Background:**

Preparing meals from raw ingredients has been linked to healthier diets, while developing cooking skills in childhood may foster lifelong healthy eating habits. In its 6th round (2021–2023), the World Health Organization (WHO) European Childhood Obesity Surveillance Initiative (COSI) added questions regarding food preparation skills.

**Methods:**

Data from 19,736 participants across eight countries were analysed. Multilevel linear regression models examined the relationship between food preparation skills practiced at home and school and daily vegetable intake, accounting for individuals nested within schools. Logistic regression was used to assess associations between experience of these skills and obesity risk. Minimally adjusted models included random intercepts for schools, while fully adjusted models also controlled for child sex and mother’s education level.

**Results:**

Increased experience of food preparation skills at home was associated with a small increase in daily fruit and vegetable intake; each one-point increment on the skills scale corresponded to a 0.09-point rise on a 5-point ordinal measure. Overall, food preparation experience at home was associated with a marginally higher odds of having obesity (OR = 1.02, 95% CI: 1.01, 1.03). Notably, experience of peeling skills was associated with lower odds of having obesity (OR = 0.84, 95% CI: 0.82, 0.86). Additionally, significant sex differences were observed: girls were more likely than boys to engage in tasks such as washing, mashing, peeling, and chopping, whereas boys more frequently reported involvement in weighing and measuring.

**Conclusion:**

Experience of food preparation skills, especially those practiced at home, is modestly associated with increased dietary intake of fruit and vegetables. Although most food preparation skills were associated with a slight increase in obesity risk, experience of peeling was linked to a 15% reduced risk. The observed sex differences in food preparation skills underline the need for targeted educational strategies. Further prospective research is needed to determine whether promoting specific food preparation skills could support healthy dietary behaviours and weight in children.

**Study registration:**

Open Science Framework https://osf.io/nfd6m/ prospective registration on 12th December 2024.

**Supplementary Information:**

The online version contains supplementary material available at 10.1007/s00394-026-03928-6.

## Background

Based on evidence, it is understood that cooking meals from scratch using raw ingredients is linked to a healthy diet and that children can learn food preparation skills during childhood to provide healthy meals and build healthy eating habits [1–3]. The World Health Organisation (WHO) Childhood Obesity Surveillance Initiative (COSI) has consistently shown sex and age differences for children’s obesity prevalence and that there are large differences across countries in the European region [4, 5]. In high-income countries, childhood obesity is often associated with lower socio-economic status and in low and middle-income countries higher socio-economic status is associated with increased obesity prevalence [6]. Little is known about the factors that may help to explain some of these differences, though variability in education, food quality consumed and income may contribute to these inequalities [6, 7].

There are no known studies that have investigated any association between food preparation skills and obesity prevalence in children aged 6–9 years. Mejean et al. (2018) found that women in France who prepared meals from scratch had 1.32 (95% CI = 1.08, 2.32) times the odds of not having obesity over a 5-year period compared to those who did not prepare meals from scratch [8]. Pelonha et al. [9] found that both sharing meal preparation responsibility and high self-efficacy in using fruits, vegetables and seasoning reduced the odds of having overweight or obesity in undergraduate students in Brazil. Sharing meal preparation responsibility reduced the odds of having overweight or obesity by 56% compared to preparing meals alone (adjusted odds ratio (AOR) = 0.44; 95% CI = 0.26, 0.74) and high self-efficacy in preparing fruits and vegetables lowered the odds by 68% (AOR = 0.32; 95% CI = 0.11, 0.95). Arslan et al. [10] found that healthier eating behaviours were associated with higher food and cooking skills in adults with overweight or obesity. For our study, we wanted to explore younger children who are helping with the preparation of family meals at home in relation to fruit and vegetable intakes and association with risk of having obesity.

The WHO Regional Office for Europe established the COSI for routine monitoring of the policy response to the emerging obesity epidemic, to measure trends in overweight and obesity in children aged 6.0–9.9 years and to allow intercountry comparisons [11]. In 2021, additional questions about children’s food preparation skills practised at school and at home were added to the COSI 6th Round of questions in the survey [12]. The aim of the present study is to explore whether having stronger food preparation skills (practised in school through nutrition education and at home in the preparing of family meals) is associated with higher fruit and vegetable intake and lower risk of having obesity in children aged 6–9 years.

### Objectives

The research objectives for the study were to answer the following questions:

1. Are higher levels of food preparation skills practiced at school associated with a lower risk of having obesity in children aged 6–9 years?
2. Are higher levels of food preparation skills practiced at home associated with a lower risk of having obesity in children aged 6–9 years?
3. Are higher levels of food preparation skills practiced at school associated with higher intake of fruit and vegetables in children aged 6–9 years?
4. Are higher levels of food preparation skills practiced at home associated with higher intake of fruit and vegetables in children aged 6–9 years?
5. Do attributes of the child, family and country moderate any associations?
6. Do girls have higher levels of food preparation skills practiced at home than boys?

The above research questions are based on the pre-registered study design at Open Science Framework on 12th December 2024 [13].

## Methods

The analysis used data collected during the COSI round 6 survey and reporting of the results followed the guidance from the Strengthening the Reporting of Observational Studies in Epidemiology (STROBE) statement [14].

The COSI survey data for round 6 was collected between 2022 and 2024, involving 37 countries from the WHO European region. The scope of the COSI survey is to include measurements from child participants aged 6–9 years. The WHO COSI protocol specifies that the effective sample size should be at least 2,800 children per target age group and that the total number of children approached should exceed these minimum thresholds [11]. Sampling design used in the COSI round 6 varied by country. The WHO Regional Office COSI team estimated sampling weights, using a standardized approach that accounted for the sampling design used in each country.

Child participants were recruited according to the published methodology and implementation processes for COSI [15]. Countries chose whether to undertake data collection online, paper or both. For paper versions, parents were provided with a sealed envelope to return it to school to minimise potential reporting bias. The full list of questions in the survey are available in the Data Collection Procedures for Round 6 [12].

In preparation for the round 6 data collection, additional new questions for countries about food preparation skills were added to the School Record Form (shown at Appendix 1) and the Family Record Form (shown at Appendix 2). The new food preparation skills were optional for countries to implement in their country surveys. Eighteen countries chose to add in the new food preparation skills questions to the School Record Form and the Family Record Form for round 6. The countries that included the additional optional questions about food preparation skills were subsequently invited to participate in the current study, with recruitment from September 2024 to December 2024. Nine countries expressed an interest to participate but one did not meet the eligibility criteria as none of the optional data about food preparation skills was collected. The eight participating countries in the study were: Bulgaria, Denmark, Germany, Hungary, Kazakhstan, Lithuania, Republic of Moldova and Republic of North Macedonia. In two of these, only smaller geographical data collections were conducted: in Germany only the federal state of Bremen participated and in Kazakhstan data was collected only in the city of Almaty. Of the eight countries, four did not meet the minimum effective sample size specified in the WHO COSI protocol, two countries nearly achieved it, and two countries exceeded it.

### Child record form

The child measurements were undertaken by trained examiners and followed the COSI protocol procedures [12]. Before weighing, children removed shoes, socks and heavy clothing (coat, jacket) wallets, mobile phones, key chains, belts and other objects. Weight was measured in kilograms and recorded to the nearest 100 g unit. Height was measured in centimetres and readings taken to the last completed 1 mm. The BMI-for-age z-score was calculated to indicate how a child’s Body Mass Index (BMI) compares to that of a reference population matched by age and sex [16]. From the child BMI-for-age z-score, we computed an additional binary variable having obesity or not using the WHO definition of greater than two standard deviations.

### School record form

The school record form was completed by the school principal (headmaster or headmistress), or the teachers of the sampled classes. Information was documented on the location of the school, the number of children registered and measured per sampled class, those who refused to be measured and those who were absent on the measuring day. Nutrition Education information was collected as a categorical variable. Schools were asked: ‘tell us what type of nutrition education your school provides’ and had four options: ‘healthy eating information’, ‘tasting of fresh fruit and vegetables’, ‘learning food preparation skills (e.g. weighing, grating, mashing, washing, chopping, peeling, measuring)’ and “no nutrition education”. We used three of these categories for descriptive statistics and selected the option ‘learning food preparation skills’ as a binary predictor variable for inferential statistics. See Appendix 1.

### Family record form

For fruit and vegetables daily intake, parents were asked: ‘Over a typical week, how many portions of fresh fruits and/or vegetables does your child eat on a typical day?’ and had 5 options to select (none, less than one portion per day, 1 to 2 portions per day, 3 to 4 portions a day, 5 or more portions per day). Parents were asked: ‘tell us about the food preparation activities that your child helps with at home’ and had seven items to select (weighing, grating, mashing, washing, chopping, peeling and measuring). Responses were binary; yes or no for each skill. See Appendix 2. Parents were asked: ‘what is the highest level of education that you or your spouse or partner has completed?’ and had 5 options from Primary education or less to Master’s or Doctoral level using combinations of levels on the International Standard Classification of Education (ISCED) framework [17].

### Data analysis

To build the model, variables were selected from the COSI survey data that are related to the experience of interest (food preparation skills practised at home) and the outcome of child BMI for age z-score. This is based on previous work on the moderators, confounders and competing experience for mapping childhood obesity using the software package, Dagitty [18]. The authors created a causal inference map (supplementary material) to highlight the variables used from the COSI survey data to include in the statistical analysis plan [18–21]. These were included in the pre-registered study in Open Science Framework [13]. A list of the variables is available at Appendix 3.

There were substantial amounts of missing data for several variables that we initially intended to include as confounders in the model: Centred Group Mean of play at weekends (14.2% missing), Group Mean Earnings (46.0% missing), and M6 Minutes per week of physical education lessons (11.6% missing). Given the extent of missingness, particularly for earnings, these variables were not included in the final adjusted models. Of all the potential variables in the COSI dataset that could be used to control for confounding, mother’s education was the most complete. Independent predictor variables included nutrition education in schools (information, tasting fruit and vegetables, food preparation skills) and food skills practised at home (weighing, grating, mashing, washing, chopping, peeling, measuring). A Total Score Food Skills at Home (scored 0–7) was computed as a predictor for the outcome variable portions of fruit and vegetable portions per day. This variable was then group mean centred to remove between-group differences and allowed a focus on individual differences [22, 23]. Confounding variables used were child’s sex and mother’s education level. We computed a group mean centred predictor variable in SPSS for mother’s education as a confounding factor for use in multilevel modelling. The dependent variables were fruit and vegetable portions eaten per day and having obesity or not.

#### Statistical methods

Initial characterisation and data quality checks were performed for the variables to be included in the analysis [24]. A primary model for multilevel linear regression was used to assess the overall impact of food preparation skills and nutrition education on fruit and vegetable intake, accounting for the hierarchical structure of the data (individuals at level 1 nested within schools at level 2) [23, 25]. To explore whether specific food skills were differently associated with the outcome, we ran secondary models for each food preparation skill, since simultaneous inclusion of all skill domains risks multicollinearity, potentially obscuring associations. We considered Bonferroni adjustment for multiple comparisons but, given the correlation among skill domains, applied a false discovery rate approach as a more statistically appropriate and widely accepted method for controlling multiple comparisons [26, 27].

Whilst the ordinal variable for daily fruit and vegetable intake does not have equal intervals, in practice 5-point ordinal variables are often treated as interval with even spaced response options [28] and a linear approach is often recommended when there are 5 or more categories for an ordinal variable [23, 29–32]. We considered using ordinal logistic regression; however, this approach relies on the proportional odds assumption, which requires the association between food preparation skills and the odds of being in a higher intake category to be constant across all thresholds. As this assumption is not necessarily less restrictive than treating the categories as approximately continuous, and because ordinal models estimate a different quantity (odds of being in a higher category rather than intake level), we retained the linear specification in line with common practice for FFQ‑derived measures. We did not use an additional country level 3, as there were a small number countries [23, 33], especially as it is not recommended to run multilevel models with three levels in SPSS with large datasets [23].

**Fig. 1:**
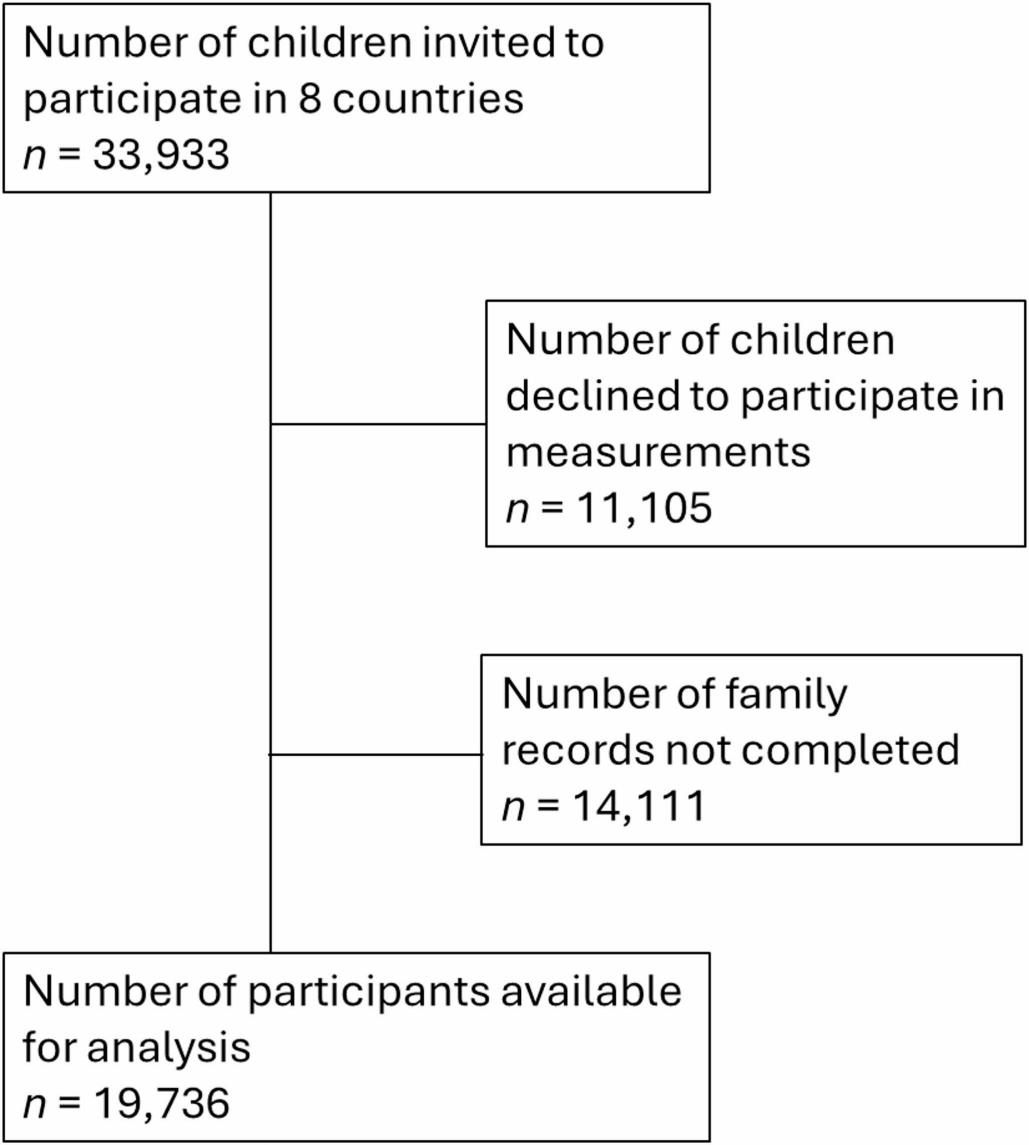
Study participant flow chart aligned with STROBE

Multilevel binary logistic regression was used to evaluate the likelihood of having obesity based on food preparation skills at home and nutrition education (which includes food skills) at school exposure. Primary analysis used a total score for food skills at home, group-mean centred, whilst secondary analysis explored whether particular food skills had different associations with the outcomes. Minimally adjusted models include random intercepts for schools, while fully adjusted models also controlled for mother’s education level and sex. All pooled data analysis across countries included a weighting factor to account for the difference between country sample sizes (Fig. 1).

## Results

In total, 19,736 child participants from eight countries were included in the study. A summary of child descriptive statistics for the final included sample, food skills experience at home and obesity prevalence by country is shown at Table 1.

**Table 1:** Summary of child descriptive statistics for sample size, food skills experience and obesity prevalence by country

		Bulgaria	Denmark	Germany (Bremen)	Hungary	Kazakhstan (Almaty)	Lithuania	Republic of Moldova	North Macadonia	Total
*Child’s sex*										
	Boys	1566	248	574	2,270	836	1,601	1,475	1,300	9,870
	Girls	1541	242	594	2,207	864	1,718	1,419	1,281	9,866
	Total	3,107	490	1,168	4,477	1,700	3,319	2,894	2,581	19,736
*Child’s age*										
	Age 6	0	189	0	1,221	1	37	155	333	1,936
	Age 7	3,107	275	190	2,110	295	2,394	2,434	2,200	13,005
	Age 8	0	4	602	1,068	875	884	304	41	3,778
	Age 9	0	0	364	67	488	0	1	5	925
*Food skills at home*										
	Weighing	18%	63%	57%	39%	14%	29%	11%	24%	29%
	Grating	29%	58%	38%	15%	26%	42%	31%	23%	28%
	Mashing	56%	47%	31%	51%	23%	29%	27%	58%	42%
	Washing	83%	74%	70%	33%	73%	73%	76%	52%	61%
	Chopping	42%	81%	79%	47%	46%	72%	59%	28%	53%
	Peeling	45%	80%	67%	47%	60%	46%	54%	32%	49%
	measuring	19%	66%	54%	90%	16%	26%	15%	31%	43%
*Obesity prevalence*										
	Boys	17%	5%	15%	14%	9%	10%	8%	17%	12%
	Girls	14%	4%	7%	11%	4%	8%	7%	12%	8%
*Portions of fruit and vegetables per day*										
	None	1%	0%	1%	2%	2%	1%	2%	3%	2%
	Less than one portion	19%	8%	7%	18%	29%	16%	22%	29%	20%
	1 to 2 portions	60%	58%	53%	64%	57%	65%	61%	56%	61%
	3 to 4 portions	17%	28%	32%	14%	10%	16%	12%	9%	15%
	5 or more portions	3%	6%	6%	2%	1%	1%	3%	3%	2%

### Nutrition education

**Fig. 2:**
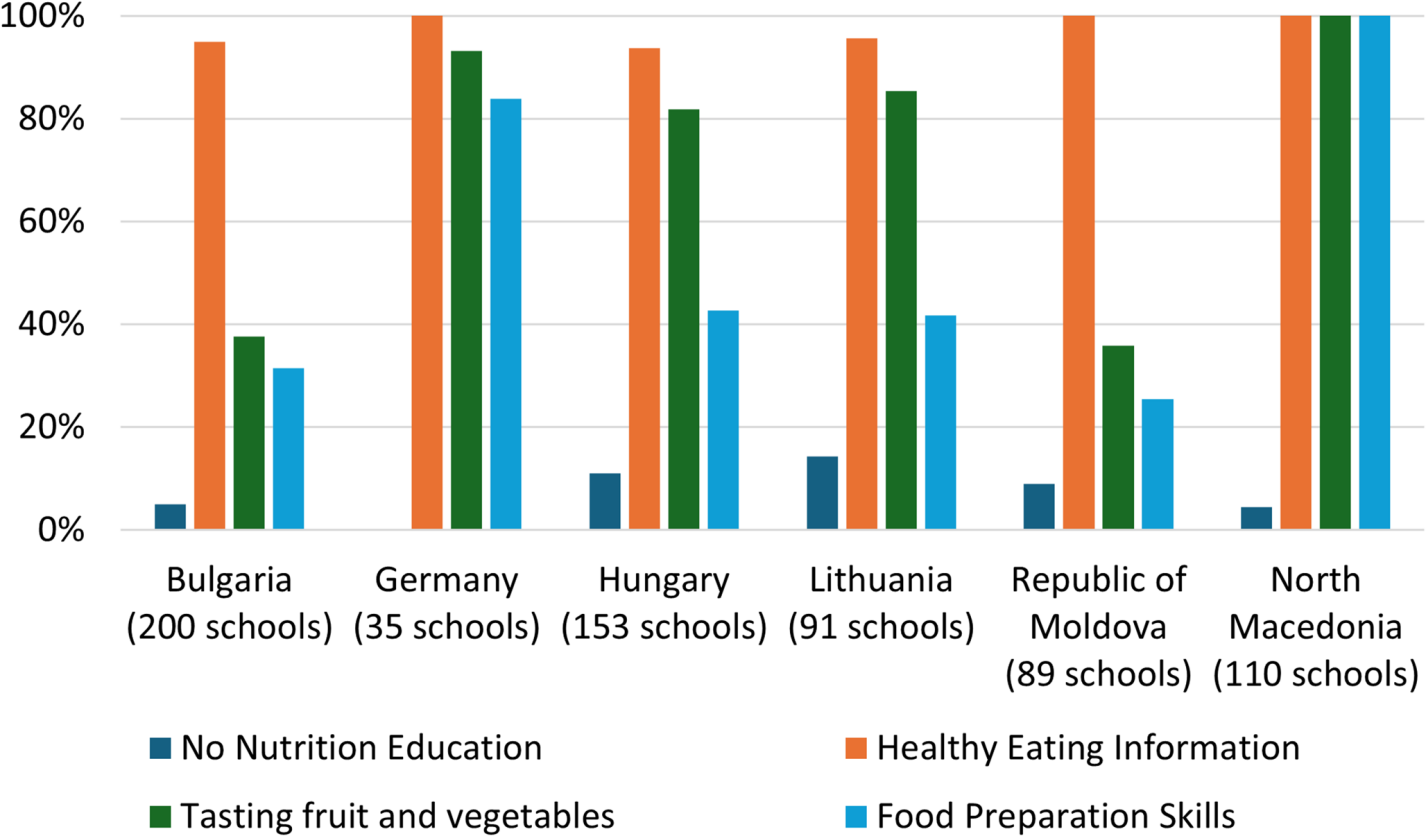
Types of nutrition education in six schools

Six out of eight countries collected data using the optional questions about type of nutrition education in schools. The results are shown in Fig. 2, indicating a wide variability of provision across countries. We noted an anomaly in the data for North Macedonia, where all schools reported that their nutrition education included tasting fruit and vegetables, healthy eating information and food preparation skills but a small number also said that there was no nutrition education. Similarly, Republic of Moldova schools all reported that they provided health eating information, but a small number of schools also said that they did not provide any nutrition education. This may be due to a misinterpretation or translation variations for the words ‘nutrition education’ in the COSI survey.

### Food preparation skills and portions of fruit and vegetables

The multilevel linear regression analysis showed that children’s engagement in food preparation skills *at home* was significantly associated with their fruit and vegetable consumption, with each one-unit increase in food preparation skills above the group mean associated with a 0.09-point rise in daily portions of fruit and vegetable consumed using a 1–5 ordinal scale (95% CI: 0.09, 0.09, *p* < 0.01). Following established guidance supporting the treatment of ordered categorical outcomes as approximately continuous in regression modelling [29, 31, 32], this change translates to roughly 0.65 extra portions over a week (0.09 portions / day x 7 days) and should be interpreted as an approximate illustration rather than a precise estimate, given that the underlying categories are not evenly spaced. This interpretation offers a practical sense of the small dietary difference linked to higher food preparation skills, expressed in units meaningful for public health discussion. See Table 2.

Subsequent models analysed if there was an association using individual food skills as predictors. The results showed that all the skills experience at home had an association with fruit and vegetable portions. Mashing had the greatest effect with 0.19 portions per day, equating to approximately an additional 1.33 portions of fruit and vegetables consumed per week (0.19 portions / day x 7 days) for children who practise mashing at home in the preparation of family meals. (B = 0.19, 95% CI = 0.19,0.2, *p* < 0.001). This is a small effect but larger than for the Total Score Food Skills at Home.

Multilevel linear regression modelling was used to explore if there was an association between food preparation skills provided in nutrition education *at school* and portions of fruit and vegetable intake but no significant results were found. See Table 2.

**Table 2:** Food skills experience at home and school and association with daily fruit and vegetable portions

	Model 1 unadjusted ^b^					Model 2 adjusted ^c^				
	95% confidence interval				ICC ^f^	95% confidence interval				ICC ^f^
	Estimate	Lower	Upper	Sig		Estimate	Lower	Upper	Sig	
Food skills at home ^a d^	0.099	0.096	0.101	< 0.001	0.243	0.093	0.09	0.096	< 0.001	0.248
Weighing ^d^	0.158	0.149	0.167	< 0.001	0.235	0.147	0.138	0.156	< 0.001	0.24
Grating ^d^	0.179	0.169	0.189	< 0.001	0.232	0.173	0.163	0.183	< 0.001	0.237
Mashing ^d^	0.206	0.197	0.214	< 0.001	0.239	0.194	0.185	0.203	< 0.001	0.244
Washing ^d^	0.185	0.176	0.194	< 0.001	0.231	0.167	0.158	0.177	< 0.001	0.237
Chopping ^d^	0.181	0.173	0.19	< 0.001	0.233	0.175	0.167	0.184	< 0.001	0.238
Peeling ^d^	0.111	0.103	0.12	< 0.001	0.236	0.098	0.09	0.107	< 0.001	0.241
Measuring ^d^	0.054	0.042	0.066	< 0.001	0.24	0.061	0.049	0.073	< 0.001	0.243
Food Skills at School ^e^	0.033	-0.051	0.118	0.442	0.169	0.033	-0.053	0.119	0.449	0.175

^a^Total Score of Food Skills experience at home (0–7) Group mean centred

^b^Unadjusted = school code as random effect to allow for clustering

^c^Adjusted = model includes country mother’s education as covariate

^d^Level 1 variable - COSI Optional Family Record Form, pooled data from 8 countries

^e^Level 2 variable - COSI Mandatory School Record Form, pooled data from 6 countries

^f^ICC = Intraclass Correlation

### Food preparation skills and obesity risk

The primary model using multilevel logistic regression analysis showed that overall, using the Total Score Food Skills at Home (0–7), children’s experience of practicing food skills at home by helping to prepare family meals was weakly associated with increased odds of having obesity. The odds ratio was 1.02 (95% CI = 1.01, 1.03), showing increased odds of having obesity of 2.3% for each additional point scored in total score food skills at home. Multilevel binary logistic regression modelling, accounting for clustering at the school level using random effects, was used to explore if there was an association between food preparation skills provided as part of nutrition education in school and having obesity among children aged 6 to 9 years. The analysis showed that the odds of having obesity compared to not having obesity was 11% higher among children attending schools that provided food skills education, compared to those in schools without this type of nutrition education. (OR = 1.11, 95% CI = 1.08,1.14), see Table 3.

In the secondary analysis, multilevel logistic regression analysis showed that some food preparation skills (e.g. peeling) were associated with lower odds of having obesity in children aged 6–9 years and other skills increased the odds of having obesity (e.g. weighing, grating, mashing, chopping, washing). The adjusted odds ratio for peeling was 0.85 (95% CI: 0.83, 0.87), indicating the children who engage in peeling food ingredients were 15% less likely to have obesity compared to those children who did not engage in peeling. See Table 3.

**Table 3:** Food skills experience at home and school and risk of having obesity

		Model 1 unadjusted ^b^						Model 2 adjusted ^c^				
		95% CI				ICC ^f^		95% CI				ICC ^f^
		Odds Ratio	Lower	Upper	Sig			Odds Ratio	Lower	Upper	Sig	
Food Skills at Home ^a d^	*n*	1.012	1.004	1.021	0.003	0.216	*n*	1.023	1.014	1.032	< 0.001	0.225
Weighing ^d^	13,491	1.058	1.03	1.088	< 0.001	0.217	12,869	1.039	1.011	1.069	0.007	0.225
Grating ^d^	13,491	1.093	1.061	1.127	< 0.001	0.217	12,869	1.092	1.059	1.127	< 0.001	0.226
Mashing ^d^	13,492	1.024	0.999	1.051	0.063	0.216	12,870	1.056	1.029	1.084	< 0.001	0.224
Washing ^d^	13,491	1.091	1.062	1.122	< 0.001	0.217	12,869	1.134	1.102	1.167	< 0.001	0.225
Chopping ^d^	13,491	1.132	1.104	1.161	< 0.001	0.218	12,869	1.13	1.101	1.16	< 0.001	0.227
Peeling ^d^	13,491	0.819	0.799	0.84	< 0.001	0.214	12,869	0.847	0.825	0.869	< 0.001	0.223
Measuring ^d^	13,492	0.941	0.908	0.975	< 0.001	0.216	12,870	0.959	0.924	0.995	0.026	0.224
Food Skills at School ^e^	14,197	1.124	1.098	1.15	< 0.001		13,470	1.11	1.084	1.137	< 0.001	

*n* Number of child participants included in the analysis

^a^Total Score of Food Skills experience at home (0–7) Group mean centred

^b^Unadjusted = school code as random effect to allow for clustering

^c^Adjusted = model includes child’s gender and mother’s education as covariates

^d^Level 1 variable - COSI Optional Family Record Form, pooled data from 8 countries

^e^Level 2 variable - COSI Mandatory School Record Form, pooled data from 6 countries

^f^ICC = Intraclass Correlation

**Fig. 3:**
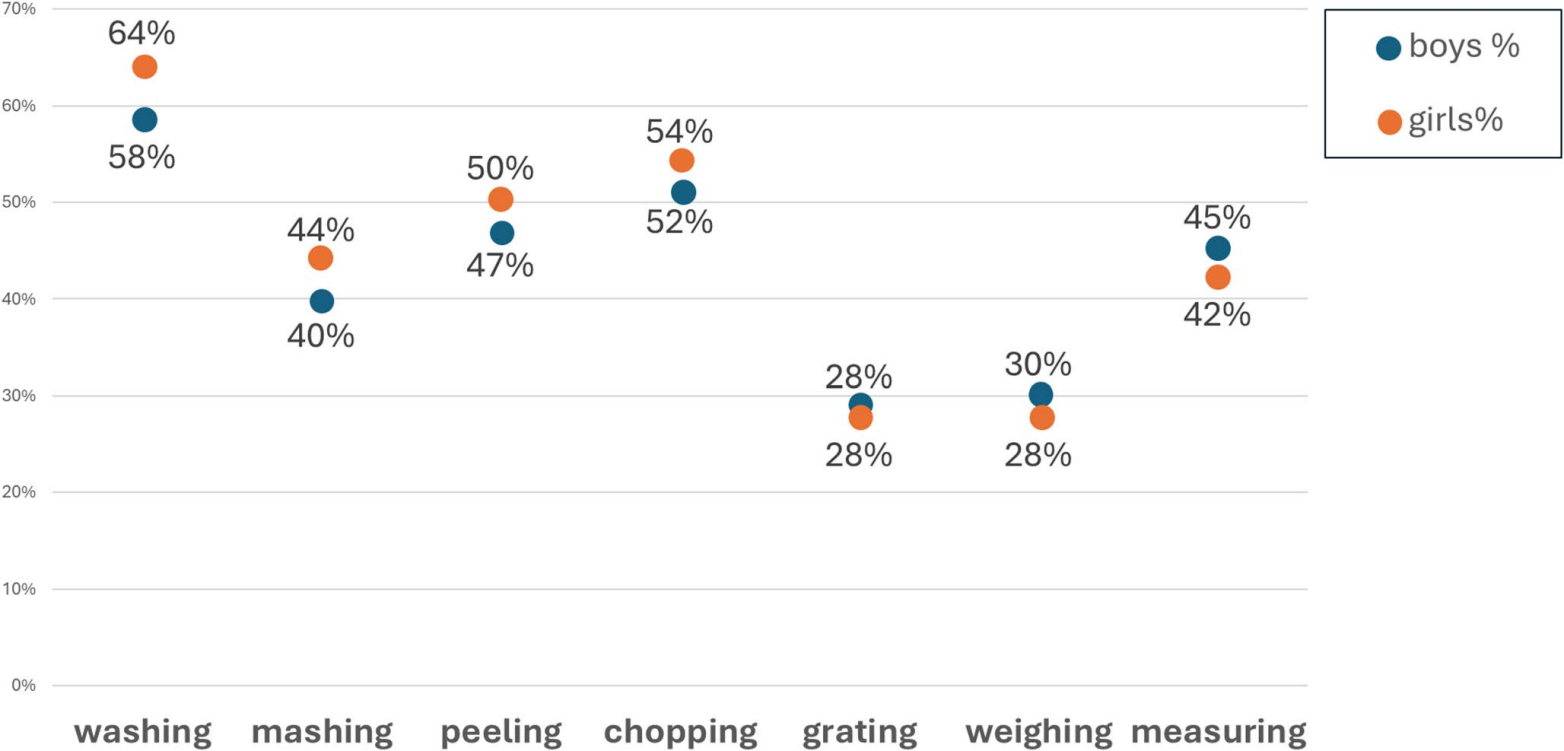
Sex differences in food preparation skills practised at home

Significant sex differences were observed for several food preparation skills, shown in Fig. 3. Girls were more likely than boys to engage in washing (χ^2^ = 45.4, *p* < 0.01), mashing (χ^2^ = 19.65, *p* < 0.01), peeling (χ^2^ = 10.6, *p* < 0.01), and chopping (χ^2^ = 6.22, *p* < 0.01). Boys were more likely to participate in weighing (χ^2^ = 7.65, *p* < 0.01) and measuring (χ^2^ = 10.74, *p* < 0.01).

## Discussion

Higher experience of food preparation skills at home was significantly associated with a small, but significant increase in portions of fruit and vegetable intake. Each additional point score in food preparation skills was associated with a 0.09-point increase on a 5-point ordinal fruit and vegetable portions daily intake scale. Because the scale is ordinal, the numerical increase does not directly translate into portion counts and the estimate should be interpreted as an approximate indication of direction and magnitude rather than a precise quantity. However, it suggests that more food skills experience in children is associated with a small but consistent increase in fruit and vegetable consumption. Overall, experience of food preparation skills at home and school is weakly associated with a slightly higher risk of having obesity. For each additional point score in food preparation skills at home this was associated with an additional 2.3% increased odds of having obesity. Skills such as washing, chopping, grating, mashing, and weighing were linked to higher odds of obesity, ranging from 4% to 13% greater odds. In contrast, peeling and measuring were associated with lower odds, corresponding to 15% and 4% reduced odds of obesity, respectively. Significant sex differences were observed in several food preparation skills. Girls were more likely than boys to engage in washing, mashing, peeling and chopping. Boys were more likely to participate in weighing and measuring.

Previous research into cooking interventions with children has focused on the outcomes of cooking confidence, increasing vegetable intake and childhood obesity reduction [3, 34–36]. However, the data analysis in the current study shows that in a larger observational study population, children who participated in more food preparation skills experience at home had a slightly higher odds ratio of having obesity. The cross‑sectional design means that reverse causation is possible; children with higher weight or appetite traits may be more involved in food preparation, and therefore the direction of association cannot be determined. Children already living with obesity, may have food-seeking appetite behaviours, and are therefore more interested in food preparation activities at home [37, 38]. Chopping and mashing may increase palatability and easy consumption of foods, potentially leading to greater intake. Intrapersonal and external influences such as behaviour, biology, cognition, hedonics and traits may all play a part in children’s appetite self-regulation and interest in being around food [39]. In addition, parents self-reported data on children’s food preparation activity may include the preparation of high-calorie meals. The association between peeling and lower odds of obesity should be interpreted cautiously, as the mechanism is unclear and may be spurious; although peeling was modestly associated with higher fruit and vegetable intake, this does not establish a causal pathway, and replication in prospective or experimental studies is needed to clarify whether this skill has any meaningful behavioural or dietary implications.

Sex differences in cooking and food preparation skills in this age group have been previously found in cooking intervention studies in the United States of America [40–42]. The present findings extend this evidence by examining differences in specific types of food preparation skills, particularly routine versus precision tasks. Girls were significantly more likely to engage in routine tasks such as washing, mashing, peeling and chopping, whereas boys were more likely to engage in weighing and measuring, which may be perceived as more precision‑based skills. These patterns are consistent with gender roles promoted in early childhood and support the view that cooking behaviours are socially constructed through cultural norms, with cooking often described as one of the most gendered household tasks [43, 44].

Broader sex differences in cooking skills, food literacy, fruit intake and obesity prevalence in children are widely recognised [40, 45–47] and the current findings align with this literature. Cultural dimensions such as dietary norms and thinness / overweight perception as a potential mechanism and to partially explain and understand differences, for example a drive for thinness in preadolescent and adolescent girls [48, 49]. Candler et al. found that 24% of the variance in thinness in girls was at country level after adjusting for individual covariates and highlighting the influence of socioeconomic and cultural contexts [50]. Motor skill development may also play a role, as girls in this age group have been shown to have more advanced motor skills than boys, potentially contributing to differences in perceived cooking competence in the COSI study [2]. However, it remains unclear whether sex differences in motor skills for cooking activities in this age group reflects biological developmental variation or different exposure to these skills arising from gender norms.

One explanation of why school nutrition which includes food skills is not associated with increased fruit and vegetable intake is that many countries in the current study did not include experience of vegetable-focussed food education in the curriculum. It is useful to compare the nutrition education policy of two countries that are outside the current study for contrast: Japan and Finland. For example, the Food Education Program in Japan is a nationally mandated initiative that integrates vegetable preparation into school nutrition education [51] and this is aligned to the school meal program [52]. There is a strong emphasis on values within the ‘Shokuiku’ (Food education and nutrition), which was highlighted as an important contextual factor in a review of school food and nutrition policies by the WHO [7]. Japan has a strong nutrition education policy linked to school meals and has an obesity prevalence of 4.4% (95% CI 3.4, 5.5) for children aged 5–19 years, compared to the world obesity prevalence for this age group of 8.2% [53]. In Finland, the home economics curriculum emphasizes that vegetable preparation is part of sustainable food education. The entire school community is engaged in school meals with student participation assisting in school canteens as part of the curriculum [54]. However, recent data from round 6 shows that the obesity prevalence for children aged 7–9 years in Finland is 13% [5].

In 2022, Smith et al. undertook a policy analysis of primary school curriculums in 11 countries on food literacy, and found that food preparation skills were most comprehensively taught in Slovenia, Iceland, Sweden, Scotland and Norway [55]. North Macedonia, Germany and Denmark have participated in case studies for school meals, showing that there are national standards for school food but no official monitoring and evaluation to ensure compliance [56–58]. The Republic of Moldova has national standards for school food in place, and this is monitored by the National Agency for Public Health and by the Ministry of Education by entering data on the nutrition of children in early education institutions (for children under 7 years) using the Education Management System [59]. Currently school meals from grades 1 to 4 are free in the Republic of Moldova and this has been extended to children up to grade 9 from September 2025 [60]. In Lithuania Ministerial order on the organization of catering at schools was implemented from 2010; the ‘Swedish table model’ was implemented in Kaunas district since 2013 enhancing school kitchen equipment and encouraging student self-service and this showed an increase in vegetable and fruit intake in primary aged children. Preschoolers, 1 and 2 grade children in Lithuania since 2021 receive a free lunch, and the policy on school meals is that it must be nutritious, hot food, cooked on the same day [61]. In Bulgaria, some basic aspects of nutrition are studied in different subjects, but they are not included in a separate subject in health education. In most schools, extracurricular activities or projects related to a healthy lifestyle are carried out, including basic aspects of nutrition. Sometimes culinary practices are included in such projects, but there are no regulated classes for food preparation skills. In Hungary, policies have focussed more on the regulation of food and drink for young people and promoting water consumption over sugar-sweetened beverages in schools [62–64].

In Cyprus, there has been a recent emphasis on promoting the Mediterranean diet with some studies showing that programmes involving cooking skills following mediterranean diet recipes was associated with increased vegetable intake in children [65]. It has previously been recognised that the amount of cooking skills hours in primary school is important, with at least 6 h or more needed to make an impact on vegetable intake in children [66]. It is beyond the scope of the COSI survey to collect detailed information on the amount of food preparation skills experience at school. However, it is likely that contextual factors such political stability, public health policy, school meal standards and monitoring will vary a lot between countries and that this will also impact on the amount and type of food skills experience in schools. Furthermore, as the study is cross‑sectional, reverse causation is possible, whereby food skills education may be present in schools that already have higher levels of overweight and obesity.

It is possible that food preparation skills experience at home is also associated with children and families who like food and enjoy both cooking and eating it. Although home cooking is often associated with healthier eating, it may also involve practices such as frequent use of processed, energy dense ingredients, high fat cooking methods, or large portion sizes. When parents prepare food with children, the activity may include baking or sweet treats, which supports family bonding and well-being but may not contribute to healthier dietary intake [67]. Children who helped with the preparation of family meals were likely to eat slightly more fruit and vegetable portions than those who did not help and had a slightly higher risk of having obesity. However, because these data are cross‑sectional and drawn from a surveillance system, the direction of these associations cannot be determined, and they should not be interpreted as evidence of a causal effect of food preparation skills on weight status. Clearly risk of obesity is highly linked to a gene and environment interaction [68, 69] and so a focus on cooking skills presents one aspect of the child’s environment which is modifiable and therefore worth exploring further which specific food preparation skills might enhance dietary quality in children. Future prospective cohort studies or randomised interventions will be required to establish whether enhancing specific food preparation skills has a causal impact on children’s fruit and vegetable intake or weight status.

### Strengths and limitations

A strength of the study is the large sample size from a range of countries and a well-established protocol for data collection from the WHO COSI team including child measurements. This is the first time this data has been used to examine the relationship between food preparation skills and obesity risk for this age group with a large dataset.

A limitation of the study is the cross-sectional design. Whilst a Directed Acyclic Graph (DAG) was used to model causal structures for confounding variables and pathways in childhood obesity, cross-sectional studies only capture data at one point in time and are therefore this study is limited in the ability to identify causal relationships. Additionally, due to missing data for some of the optional questions it was not possible to add a few confounding variables. Some variables with potential causal links to childhood obesity prevalence such as sleep quality and other health factors could not be included in the analysis, either because the data was not collected in the survey, or because there was too much missing data for this to be successfully computed in the multilevel linear regression models. Imputation for missing data was considered, but for some variables (for example family income) it was not deemed to be missing at random, and therefore not appropriate for imputation [23].

A further limitation is that the COSI survey does not incorporate validated measurement tools for many research-relevant variables. For instance, children’s food skills were assessed through parent reports in the Family Record Form, which may be less reliable than child self-reports.

## Conclusions

Overall, in children aged 7–9 years the COSI round 6 survey shows that one in ten children is living with obesity in Europe [5]. Ending childhood obesity remains an important priority for global public health and data from COSI plays an important role in both monitoring progress and informing policy-makers. This study has shown that experience of food preparation skills, especially those practiced at home, is modestly associated with increased dietary intake of fruit and vegetables. Surprisingly, most food preparation tasks were associated with a slight increase in obesity risk, although this was a small effect. Experience of peeling was linked to 15% reduced odds of having obesity. The observed sex differences in food preparation tasks emphasises the need for a review of nutrition education curricula in schools. Prioritizing skills such as peeling and mashing may increase fruit and vegetable intake in children. However, these associations are derived from cross‑sectional data, may be influenced by unmeasured confounding, and should not be interpreted as causal. Future prospective cohort studies or randomised interventions will be required to establish whether enhancing specific food preparation skills has a causal impact on children’s fruit and vegetable intake or weight status.

## Supplementary Information

Below is the link to the electronic supplementary material.

Supplementary Material 1

Supplementary Material 2

Supplementary Material 3

Supplementary Material 4

Supplementary Material 5

## Data Availability

The code created and datasets analysed for the current study may be available upon request to the authors.
