# Supplementary material for: Food preparation skills and obesity risk in European children aged 6–9 years: a cross-sectional study using WHO COSI 2022–2024’
Source: Eur J Nutr. 2026 Feb 28;65(2):71. doi: 10.1007/s00394-026-03928-6 (PMC12950072; doi:10.1007/s00394-026-03928-6)
Supplement: Supplementary file 1 — Supplementary Material 1 [file 394_2026_3928_MOESM1_ESM.docx]

Appendix 3 Data definitions for variables and sample size

| Variable | Number | Level | Description | Values | Measurement |
| --- | --- | --- | --- | --- | --- |
| Obesity | 19,428 | Individual | Outcome variable – has obesity or not calculated from BMI-for-age z-score (≥ +2 SD from WHO growth reference) | 0 = no,1 = yes | Dichotomous |
| Daily fruit and vegetable intake | 19,398 | Individual | Outcome variable with 5 ordered categories | 1,2,3,4,5 | Ordinal |
| Group mean centred food skills at home | 13,708 | Individual | Predictor variable – group mean centred from total food skills at home. Original ordinal scale for total food skills at home scored 0,1,2,3,4,5,6, 7. | -3.9 to 5 | Scale |
| Weighing | 13,708 | Individual | Predictor variable – binary, weighs at home or not | 0 = no,1 = yes | Dichotomous |
| Grating | 13,708 | Individual | Predictor variable – binary, grates at home or not | 0 = no,1 = yes | Dichotomous |
| Mashing | 13,709 | Individual | Predictor variable – binary, mashes at home or not | 0 = no,1 = yes | Dichotomous |
| Washing | 13,708 | Individual | Predictor variable – binary, washes at home or not | 0 = no,1 = yes | Dichotomous |
| Chopping | 13,708 | Individual | Predictor variable – binary, chops at home or not | 0 = no,1 = yes | Dichotomous |
| Peeling | 13,708 | Individual | Predictor variable – binary, peels at home or not | 0 = no,1 = yes | Dichotomous |
| Measuring | 13,709 | Individual | Predictor variable – binary, measures at home or not | 0 = no,1 = yes | Dichotomous |
| Food skills at school | 14,432 | School | Predictor variable – binary, school provides practical food skills education or not | 0 = no,1 = yes | Dichotomous |
| Group mean centred mother’s education level | 18,761 | Individual | Confounding variable – group mean centred from mother’s education level. Original ordinal scale for mother’s education level scored 1,2,3,4,5. | -3.58 to 3.3 | Scale |
| Sex | 19,736 | Individual | Confounding variable – male or female | 1 = boys, 2 = girls | Dichotomous |
