# Supplementary figures and images for: Food preparation skills and obesity risk in European children aged 6–9 years: a cross-sectional study using WHO COSI 2022–2024’
Source: Eur J Nutr. 2026 Feb 28;65(2):71. doi: 10.1007/s00394-026-03928-6 (PMC12950072; doi:10.1007/s00394-026-03928-6)

# Appendix 1

# Nutrition Education questions COSI School Record Form (M7 and O12)

#
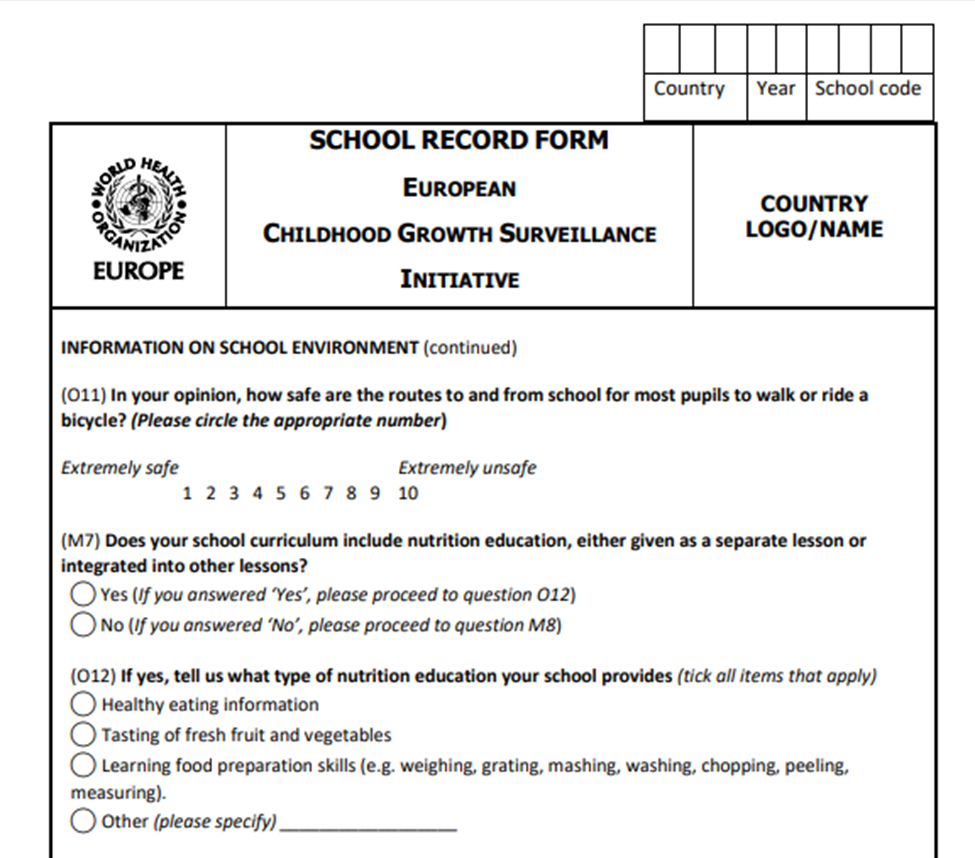

Supplement: Supplementary file 3 — Supplementary Material 3 [file 394_2026_3928_MOESM3_ESM.docx]

# Appendix 2

# Food preparation questions COSI Family Record Form

# (020 and 021)

#
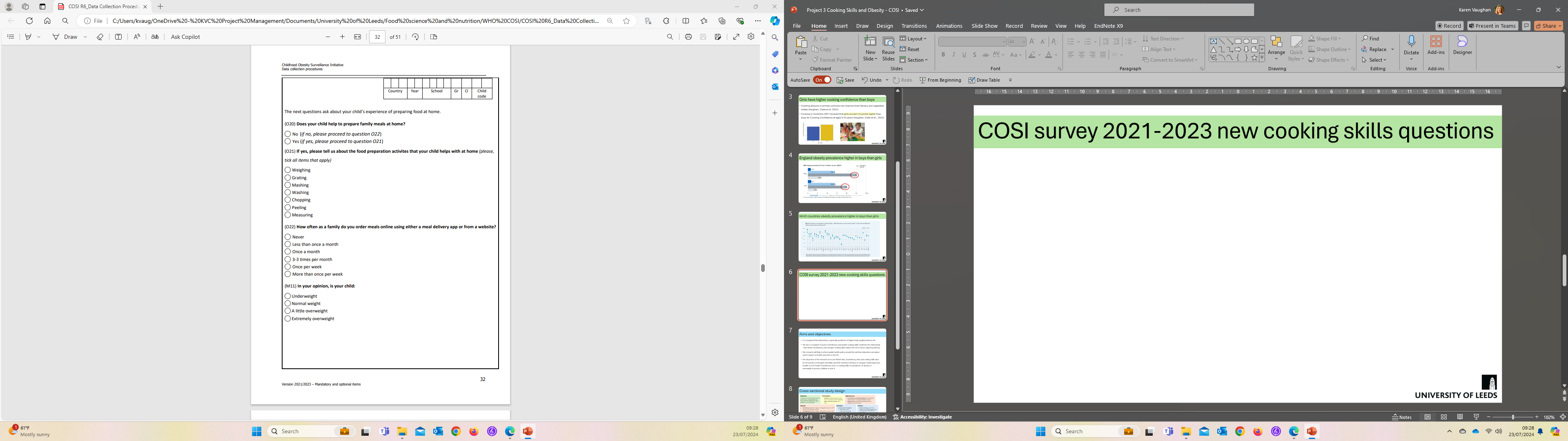

Supplement: Supplementary file 4 — Supplementary Material 4 [file 394_2026_3928_MOESM4_ESM.docx]

### Appendix 4 Directed Acyclic Graph for COSI Cooking Study

**
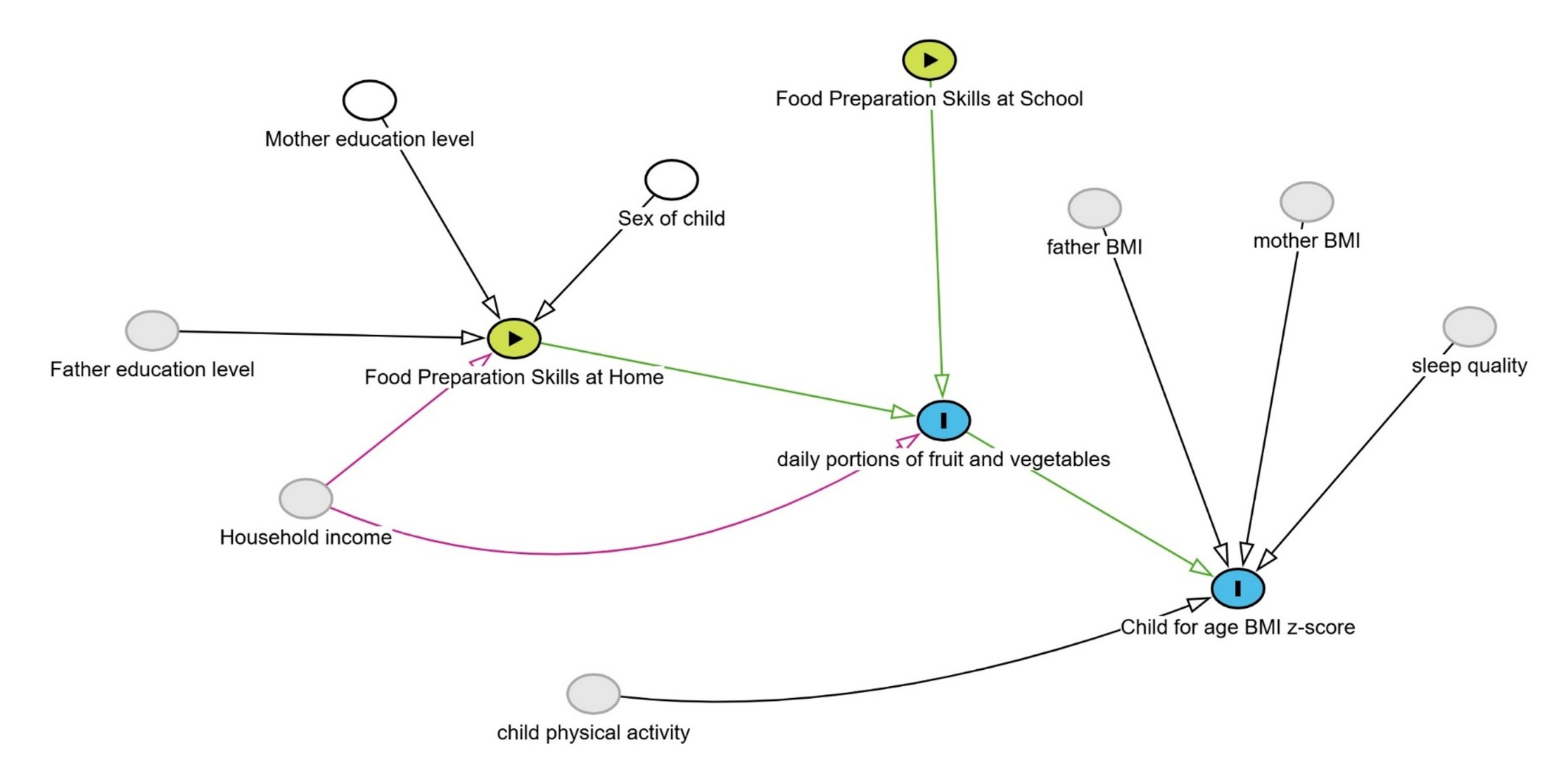
**

Supplement: Supplementary file 5 — Supplementary Material 5 [file 394_2026_3928_MOESM5_ESM.docx]
